# Genetic screening of Fabry patients with EcoTILLING and HRM technology

**DOI:** 10.1186/1756-0500-4-323

**Published:** 2011-09-06

**Authors:** Caterina Bono, Domenico Nuzzo, Giuseppe Albeggiani, Carmela Zizzo, Daniele Francofonte, Francesco Iemolo, Enzo Sanzaro, Giovanni Duro

**Affiliations:** 1 National Research Council-Institute of Biomedicine and Molecular Immunology (CNR-IBIM) - Palermo, Italy; 2 Department of Neurology - Guzzardi Hospital - Vittoria (Ragusa), Italy

**Keywords:** Anderson-Fabry, haplotype, screening, HRM, EcoTILLING

## Abstract

**Background:**

Anderson-Fabry disease (FD) is caused by a deficit of the α-galactosidase A enzyme which leads to the accumulation of complex sphingolipids, especially globotriaosylceramide (Gb3), in all the cells of the body, causing the onset of a multi-systemic disease with poor prognosis in adulthood. In this article, we describe two alternative methods for screening the *GLA *gene which codes for the α-galactosidase A enzyme in subjects with probable FD in order to test analysis strategies which include or rely on initial pre-screening.

**Findings:**

We analyzed 740 samples using EcoTILLING, comparing two mismatch-specificendonucleases, CEL I and ENDO-1, while conducting a parallel screening of the same samples using HRM (High Resolution Melting). Afterwards, all samples were subjected to direct sequencing. Overall, we identified 12 different genetic variations: -10C>T, -12G>A, -30G>A, IVS2-76_80del5, D165H, C172Y, IVS4+16A>G, IVS4 +68 A>G, c.718_719delAA, D313Y, IVS6-22C>T, G395A. This was consistent with the high genetic heterogeneity found in FD patients and carriers. All of the mutations were detected by HRM, whereas 17% of the mutations were not found by EcoTILLING. The results obtained by EcoTILLING comparing the CEL I and ENDO-1 endonucleases were perfectly overlapping.

**Conclusion:**

On the basis of its simplicity, flexibility, repeatability, and sensitivity, we believe thatHRM analysis of the *GLA *gene is a reliable presequencing screening tool. This method can be applied to any genomic feature to identify known and unknown genetic alterations, and it is ideal for conducting screening and population studies.

## Introduction

Fabry disease (FD) is a lysosomal storage disease caused by a congenital error in glycosphingolipid metabolism resulting from the deficient or absent activity of the lysosomal enzyme α-galactosidase A [1]. This enzyme is essential for the sphingolipid recycling process that occurs within cells. It is able to remove the galactose bound in the alpha position in complex sphingolipids, allowing their metabolism. The enzyme deficiency causes the interruption of the process of waste product demolition, leading to the intracellular accumulation of complex sphingolipids, especially globotriaosylceramide (Gb3) [2]. The accumulation of these products is the basis of FD symptomology that includes acroparesthesias, angiokeratoma, corneal and lenticular opacities, gastrointestinal problems and diseases of the kidney, heart and central nervous system [3-5]. The *GLA *gene (12 kb) contains seven exons (92 to 291 bp), is located on the X chromosome, and encodes a polypeptide of 429 amino acids [6].

In the literature, more than 500 mutations in the *GLA *gene have been associated with FD [7]. Of these, only 5% are localized in exons and are able to cause the formation of a truncated protein. Instead, for the most part, they involve missense mutations generating null alleles or changes in specific domains of the protein; others, such as those that occur in splicing sites, can lead to aberrant splicing and can be responsible for altered forms of the enzyme [8,9]. Polymorphisms, small deletions, and insertions in the *GLA *gene have been detected by direct sequencing [10], through single-strand conformation polymorphism analysis [11], and by denaturing high performance liquid chromatography (DHPLC) [12]. However, these investigative techniques may be limited in the analysis of large numbers of samples, so it is therefore necessary to move towards new analytical approaches which include or rely on pre-screening.

In our study, we performed genetic analyses of the *GLA *gene in 740 subjects with symptoms related to FD using two different approaches to genetic screening. The first was a method based on the enzymatic cleavage of mismatches, known by the acronym EcoTILLING (Targeting Induced Local Lesions in Genomes), originally used for studying the plant genome and adapted to researching human genetic variations [13]. We conducted these analyses using a screening strategy based on pools of 8 samples using two mismatch-specific endonucleases, CEL I (SURVEYOR Mutation Detection Kit-Transgenomic) and ENDO-1 (Serialgenetic). The second method was based on HRM which is able to detect genetic mutations based on the dissociation temperature of the two strands (Tm) and the analysis of the melting curves (LightCycler480, Roche)[14]. We applied these techniques to analyze the coding regions and intron junction regions of the *GLA *gene in order to investigate mutations in the coding regions and splicing regulatory sites. Finally, to verify the results obtained, each sample was analyzed by direct sequencing.

## Materials and methods

### DNA extraction and quantification

DNA was extracted by column extraction (GenElute Blood Genomic DNA Kit, Miniprep - Sigma Aldrich) from 740 peripheral blood samples of subjects with clinical manifestations related to FD symptomatology. The study was approved by University Hospital Ethics Committee and written informed consent was obtained from all participants.

The concentration of the DNA was determined by spectrophotometer (Victor X3 - PerkinElmer), and the concentration was adjusted to 100 ng/microliter in TE (10 mM Tris-HCl and 1 mM EDTA, pH 7.4) or water.

### Primer design

Seven pairs of primers were designed for the analyses of the seven target regions containing the seven exons of the *GLA *gene and the splicing regulatory regions flanking them, and they were obtained using the GeneTools program. The gene sequence was obtained from the site http://www.ensembl.org (ENSG00000102393). The primers used for the EcoTILLING and HRM analyses were identical but for marking with IRDye700 in forward and IRDye800 in reverse (Table 1).

**Table 1:** The seven primer pairs used for HRM analyses and the corresponding amplification fragments.

Region	Primers	Amplicon sizes	Exon sizes	MgCl_2 _conc.
Exon 1	5'-TCTTACGTGACTGATTATTGGTCT-3' 5'-CACACCCAAACACATGGAAA-3'	416 bp	254 bp	3.5 mM

Exon 2	5'-TGAAATCCCAAGGTGCCTAATA-3' 5'-GTACAGAAGTGCTTACAGTCC-3'	314 bp	174 bp	3.5 mM

Exon 3	5'-ACCTGGTGAAGTAACCTT-3' 5'-CTCAGCTACCATGGCCT-3'	349 bp	178 bp	3.5 mM

Exon 4	5'-GCTGGAAATTCATTTCTTTCCC-3' 5'-GGATGGTGAGAAGTGGTTG-3'	285 bp	91 bp	3.5 mM

Exon 5	5'-AATCTGTAAACTCAAGAGAAGGCTA-3' 5'-CTTTACCTGTATTTACCTTGAATG-3'	349 bp	161 bp	3.5 mM

Exon 6	5'-GATGCTGTGGAAAGTGGTT-3' 5'-GCCCAAGACAAAGTTGGTAT-3'	355 bp	198 bp	3 mM

Exon 7	5'-AGAATGAATGCCAAACTAAC-3' 5'-ATGAGCCACCTAGCCTTG-3'	443 bp	295 bp	3.5 mM

The primers used in the EcoTILLING protocol were identical and differentiated by their marking with IRDye700 and IRDye800 in forward and reverse, respectively.

### EcoTILLING pooling

**Figure 1:**
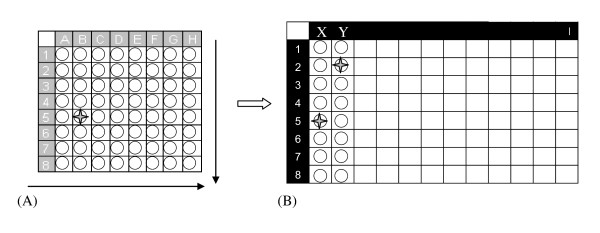
**Diagram of sample pooling - (A) For the pooling strategy, 64 samples were first allocated in an 8 × 8 grid and then mixed in pools of 8 samples each**. (B) The 16 pools obtained were arranged in a second plate and subjected to the EcoTILLING protocol. After enzymatic digestion, the identification of the pools with the same fragments permits the identification of the presence of heterozygotes and to go back to the coordinates that identify the mutated sample.

For the simultaneous analysis of a large number of samples, we chose a strategy of pooling, as described by Till et al. [15]. For a high-throughput screening, the 740 DNA samples were divided into groups of 64 samples each, arranged in 8 × 8 grids and then assembled in pools of 8 samples (Figure 1). Each pool was obtained by adding all the samples present in a row or column of the grid, repeating the procedure for the 8 rows and 8 columns of each grid. The 16 pools derived from each grid were arranged in a second plate and subjected to the EcoTILLING method. The presence of the fragments in two pools containing the same sample allowed us to trace the exact location of the sample in the grid and then to identify the subject.

Each PCR was performed in a final volume of 50 μl (Hot Start Master Mix - GE Healthcare) with 1 μM of primers, 50 ng of DNA. The amplification was performed using the following parameters: 95°C for 2 minutes, 40 cycles with 95°C for 30 minutes, 58°C for 30 minutes, 72°C for 1 minute, and a final extension at 72°C for 10 minutes.

Mixing 8 different samples ensures the formation of a heteroduplex in the presence of a mutation. The samples thus obtained were denatured and renaturated according to the following parameters: 95°C for 2 minutes, followed by a decrease in temperature of 0.5°C for 15 minutes, until 45°C. The heteroduplexes were subjected to enzymatic digestion by the endonucleases CEL I (SURVEYOR Mutation Detection Kit - Transgenomic) and, in parallel, ENDO-1 (Serialgenetics). The reaction was incubated at 42°C for 20 minutes and stopped by adding 1.5 μl stop buffer, or at 37°C for 40 minutes and stopped with 5 μl stop buffer, respectively. All reagents were supplied by the Surveyor kit (SURVEYOR Mutation Detection Kit - Transgenomic) and by the ENDO-1 Endonuclease Kit (Serialgenetics).

### Electrophoretic Separation

The fragments were separated by electrophoretic migration (DNAanalyzer 4300 - Licor) on polyacrylamide gel (KB-plus 6.5% gel-matrix - Licor). The scanning of gels subjected to electrophoresis permits the detection of the presence of mutations and their location in the analyzed fragment, deduced on the basis of the size of the fragments obtained. The gels were analyzed using the GelBuddy program in manual mode.

### PCR-High Resolution Melting (HRM)

**Figure 2:**
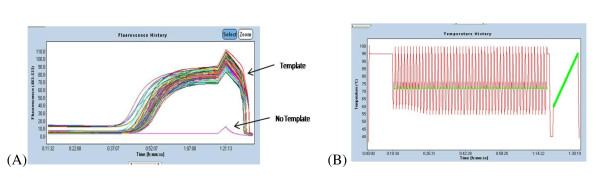
**Amplification curves - (A) Amplification curves with characteristic sigmoid pattern**. (B) Cycles of amplification, with the moments of data acquisition by the CCD camera indicated in green. The area highlighted by a solid green line represents the dissociation phase of the strands.

Genetic screening for the presence of mutations in the *GLA *gene was conducted by Real-Time PCR and subsequent HRM (Figure 2). The PCR was carried out on a final volume of 20 μl, according to the following amplification program: 95°C for 10' minutes, 50 cycles at 95°C for 10 minutes, 62°C for 15 minutes, 72°C for 18 minutes. The amplification in Real-Time PCR and the dissociation of the two DNA strands were performed in the presence of a fluorescent marker (Light Cycler^® ^480 High Resolution Melting Dye). In order to ensure standardization of the method, male subjects were analyzed by adding a wild type DNA counterpart, in a 1:1 volume ratio.

### Sequencing

To test the reliability of the results obtained, all samples were subjected to direct sequencing according to the following program: 94°C for 2 minutes, 30 cycles at 96°C 30 minutes, 58°C for 30 minutes, 72°C for 1 minute, holding at 4°C.

## Results

### EcoTILLING

**Figure 3:**
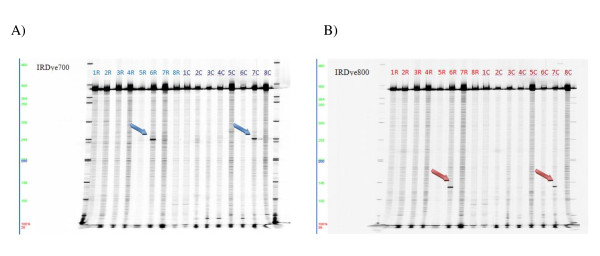
**Pooling strategy for Exon 1 of the *GLA *gene - The 16 lines represent the 16 pools obtained through the pooling strategy for rows (1R-8R) and columns (1C-8C)**. Above, PCR products undigested at 390bp, visible in both detection channels. (A) Images obtained in the 700 nm channel for the identification of fragments marked by the forward-IRDye700 primer. (B) Images obtained in the 800nm channel for the identification of fragments marked by the reverse-IRDye800 primer. The 6R and 7C pools show fragment sizes of approximately 135 bp, indicated by blue arrows (for IRDye 700), and 255 bp, indicated by red arrows (for IRDye 800). Sizes of the digested fragments indicate the position of the mutation within the amplicon, and their sum comprises the dimensions of the undigested 390 bp fragment.

The need to perform simultaneous analyses of many samples led to the adoption of a high-throughput screening strategy based on pools of 8 samples, as reported by Till et al. [15]. In this study, we have carried out genetic screening of the seven chosen target regions for the analysis of the *GLA *gene on 740 samples (Figure 3). The analysis was done by comparing two enzymes with endonuclease activity (ENDO-1 and CEL I), and their recognition efficiency and heteroduplex cut fragments were identical. The mutations and polymorphisms identified by EcoTILLING are shown in Table 2.

**Table 2:** Diagram of the identified genetic variations after enzymatic digestion with CEL I and ENDO-1 and their location.

		Region 1	Region 2	Region 3	Region 4	Region 5	Region 6	Region 7
		**Exon 1**	**Exon 2**	**Exon 3**	**Exon 4**	**Exon 5**	**Exon 6**	**Exon 7**

**Haplotype 1**		-10 C>T	wt	IVS2 - 76_80del5	wt	IVS4 -16A>G	wt	wt
		
**Haplotype 2**	Polymorphism	wt	wt	IVS2 - 76_80del5	wt	IVS4 -16A>G	wt	wt
		
**Haplotype 3**		-12G>A	wt	wt	IVS4 +68A>G	wt	wt	wt
		
		-10 C>T	wt	wt	wt	wt	wt	wt
	
	Mutations	-30G>A	wt	wt	wt	wt	wt	wt
		
		wt	wt	C172Y	wt	wt	wt	wt
		
		wt	wt	wt	wt	wt	D313Y	wt
		
		wt	wt	wt	wt	wt	wt	wt
		
		wt	wt	wt	wt	c.718_9 dellAA	wt	wt
		
		wt	wt	D165H	wt	wt	wt	wt

### High Resolution Melting

**Figure 4:**
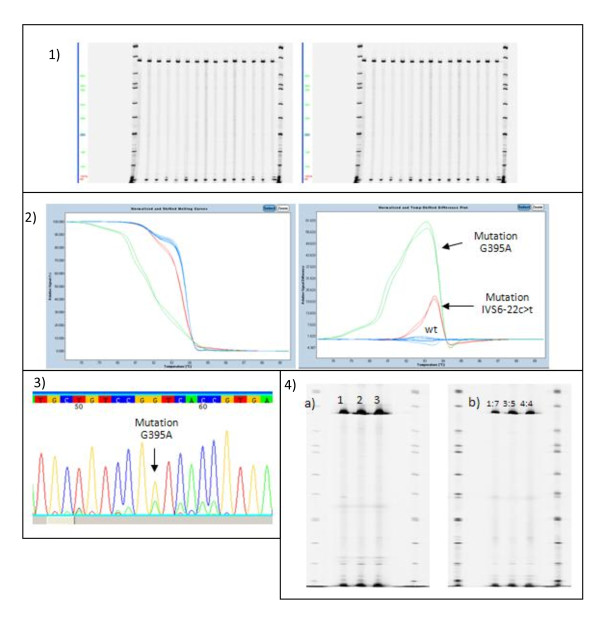
**EcoTILLING screening - Figure shows images related to the EcoTILLING screening conducted on 68 samples amplified with the primer pair for Region 7**. EcoTILLING analysis did not report the presence of some mutated samples (1) which were identified through HRM (2) and confirmed by sequencing (3). (4) EcoTILLING experiments done by varying the digestion time or the number of samples per pool did not change the results. a) Digestion times for pools with CEL I or ENDO-1. Lane 1: 15'; lane 2: 20'; lane3: 30'. Images of a single detection channel. b) Proportion variations between mutated samples and wild type.

In order to evaluate the results obtained by EcoTILLING, we conducted another screening with High Resolution Melting. The ten mutations/polymorphisms identified by EcoTILLING were confirmed, and two further gene variations (G395A; IVS6-22c>t), undetected by enzymatic digestion by the CEL I and ENDO-1 endonucleases, were identified (Table 3). The mutations which were undetected by EcoTILLING were analyzed separately, applying variations of the protocols: increases in the enzymatic digestion times or changes in analysis of the relationship between mutated and wild type samples in the pool (Figure 4).

**Table 3:** List of genetic variants found in the samples analyzed by enzymatic digestion using the CEL I or ENDO-1 endonuclease and by High Resolution Melting.

	Variation	EcoTILLING (CelI or EndoI)	HRM
**Region 1**	-10 c>t	yes	yes
	
	-12 g>a	yes	yes
	
	-30 g>a	yes	yes

**Region 2**	IVS2-76_80 del5	yes	yes

**Region 3**	D165H	yes	yes
	
	C172Y	yes	yes

**Region 4**	IVS4+68 A>G	yes	yes

**Region 5**	IVS4-16 A>G	yes	yes
	
	c.718_719 del AA	yes	yes

**Region 6**	D313Y	yes	yes

**Region 7**	**IVS6-22 C>T**	**no**	**yes**
	
	**G395A**	**no**	**yes**

The G395A mutation and the IVS6-22C>T polymorphism, identified exclusively through HRM, are shown in bold.

After direct sequencing of the 740 samples, no discrepancies were found in the HRM results (Table 4). According to the data, this technique appears to be the optimal method for pre-sequencing screening of the *GLA *gene. Indeed, the robustness of this method ensures the identification of all possible genomic variants.

**Table 4:** Diagram of the genetic variations identified after HRM and their location.

		Region 1	Region 2	Region 3	Region 4	Region 5	Region 6	Region 7
		**Exon 1**	**Exon 2**	**Exon 3**	**Exon 4**	**Exon 5**	**Exon 6**	**Exon 7**

**Haplotype 1**		-10 C>T	wt	IVS2 - 76_80del5	wt	IVS4 -16A>G	wt	**IVS6 -22C>T**
		
**Haplotype 2**	Polymorphism	wt	wt	IVS2 -76_80del5	wt	IVS4 -16A>G	wt	**IVS6 -22C>T**
		
**Haplotype 3**		-12G>A	wt	wt	IVS4 +68A>G	wt	wt	**IVS6 -22C>T**
		
		wt	wt	wt	wt	wt	wt	**IVS6 -22C>T**
	
	Mutations	-30G>A	wt	wt	wt	wt	wt	wt
		
		wt	wt	C172Y	wt	wt	wt	wt
		
		wt	wt	wt	wt	wt	D313Y	wt
		
		wt	wt	wt	wt	wt	wt	**G395A**
		
		wt	wt	wt	wt	c.718_9 dellAA	wt	wt
		
		wt	wt	D165H	wt	wt	wt	wt

## Conclusions

The use of a screening system permits the identification of samples to be subjected to sequencing, the performance of the simultaneous analysis of numerous genetic traits, and the undertaking of population studies.

EcoTILLING technology is recognized by the scientific community as a method for the identification and characterization of single nucleotide polymorphisms (SNPs) and small and large deletions (indels) [16-18]. In this study we analyzed pools of eight samples to reduce screening time and/or the cost associated with the analysis of 740 samples, identifying ten genetic variants across the seven exons and their flanking regions of the *GLA *gene. After HRM analysis, we discovered two other previously undetected mutations.

The detection of mutations with EcoTILLING shows a number of disadvantages that limit its use in analyses of human genetic diseases and in clinical medicine investigations. In particular, mismatch recognition sensitivity is reduced when mutations are present at the ends of the target sequence [15,19-23] and as the number of polymorphisms identified for each fragment increases.

The acquisition of melting profiles in HRM is not dependent on the location and number of mutations present in the analyzed fragment, but only on changes in the dissociation kinetics of the amplified fragments with respect to the wild type [24-27]. Because of its high reliability and flexibility, it is currently employed as a method of genotyping in clinical and diagnostic settings [28,29]. In genetics, this method has been applied to BRCA1/2 hereditary breast cancer [30], cystic fibrosis [31], haemophilia [32], the instability of microsatellites [33], the rearrangement of heavy chain genes in lymphomas [34], the prenatal diagnosis of B-thalassaemia [35], analysis of DNA methylation [36], the identification of bacterial species [37,38], and other applications. Considering the polymorphic nature of the *GLA *gene, we sustain that HRM analysis appears to be an ideal investigative tool which permits the search for all possible gene variations with a high level of reliability.

Finally, in the cases reported, some mutations seemed to occur in haplotype patterns, as studies of genealogical trees of the proband families confirmed. Indeed, 12% of the subjects analyzed showed polymorphisms in the promoter region of the *GLA *gene and, of these, 99% exhibited several simultaneous polymorphisms spread throughout the gene. In particular, the -10 c>t, IVS2-76_80 del5, IVS4-16 A>G, IVS6-22C>T and -12 g>a, IVS4+68 A>G, IVS6-22C>T polymorphisms occured simultaneously in 8.9% and 3.7% of the subjects, and the significance of this haplotype in FD pathology remains unknown. In a pilot study of patients with small fiber neuropathy of unknown etiology, published in August 2010, Tanislav et al. reported the presence of a complex intronic haplotype within the *GLA *gene (-10 c>t, IVS2-76_80 del5, IVS4-16 A>G, IVS6-22C>T) in four patients which is identical to the first of the haplotypes mentioned above for our subjects [39]. The -12 g>a, IVS4+68 A>G, IVS6-22C>T haplotype identified in approximately 4% of our samples has not been reported in the literature.

## Competing interests

The authors declare that they have no competing interests.

## Authors' contributions

CB drafted the manuscript and conducted EcoTILLING experiments, DN design the study and conducted HRM experiments, GA carried out the molecular genetic study, CZ performed the statistical analysis and participate in the sequence alignment, DF participate in the sequence alignment, FI participated in acquisitions of a data and interpretations of data, ES participated in acquisitions of a data and interpretations of data, GD participated in the coordination of the study obtaining the informed consent and ethics approval to conduct the study approval. All authors read and approved the final manuscript.
